# Hypoxia Limits the Growth of Bovine Follicles in Vitro by Inhibiting Estrogen Receptor α

**DOI:** 10.3390/ani9080551

**Published:** 2019-08-13

**Authors:** Lizhu Ma, Liqiang Wang, Huimin Gao, Ning Liu, Yuxin Zheng, Yan Gao, Shujie Liu, Zhongliang Jiang

**Affiliations:** 1 College of Animal Science and Technology, Northwest A & F University, Yangling 712100, China; 2 Faculty of Medical and Chemical Engineering, Xianyang Vocational Technical College, Tongyi Street, Fengxi new city, Xi’an 712100, China; 3 State Key Laboratory of Plateau Ecology and Agriculture, Key Laboratory of Plateau Grazing Animal Nutrition and Feed Science of Qinghai Province, Qinghai Plateau Yak Research Center, Qinhai University, Xining 810016, China

**Keywords:** follicle, bovine, hypoxia, estrogen receptor

## Abstract

**Simple Summary:**

It is well documented that a hypoxic environment at high altitudes decreased the fertility of female domestic animals because of embryonic or fetal losses, intrauterine growth restriction, and birth weight reduction. However, little study has been performed on for the effects of hypoxia on bovine ovary function. In this study, we found that the hypoxia plays an important role in regulating follicular survival and genes expression. Hypoxia limits the growth of bovine follicles cultured in vitro through inhibition of ERα, which could provide useful information for future studies relating to reproduction of cattle.

**Abstract:**

Female animals living in the Qinghai-Tibet Plateau have lower ovulation rates because of the hypoxic environment, however, the mechanism of hypoxia on animal follicles is unclear. In this study, the effects of hypoxia on bovine follicles were investigated using an in vitro follicular culture system. The results show that there was a significant decrease in follicular diameter from day 3 to day 6 in both hypoxia and hypoxia with estrogen (E_2_) and fulvestrant (ICI 182780) (hypoxia + E_2_ + ICI) groups, when compared with a normoxia group (*p* < 0.05). We also observed significant downregulation of ERα and *FSHR*, while upregulation of *LHCGR* expression in the hypoxia group and hypoxia + E_2_ + ICI groups compared to the normoxia group (*p* < 0.05). The expression of *IGF1* gene was significantly downregulated in hypoxia + E_2_ + ICI group when compared to the hypoxia + E_2_ group (*p* < 0.05). The expression of *HIF1A*, *ADAMTS1*, *VEGFA*, and *EDN2* were upregulated in both hypoxia and hypoxia + E_2_ + ICI groups in comparison to normoxia group (*p* < 0.05). Under hypoxic conditions, the addition of E_2_ resulted in a decrease of HIF1A protein but an increase of ERα protein in cultured bovine follicles (*p* < 0.05). In summary, hypoxia limits the growth of bovine follicle cultured in vitro through inhibition of ERα.

## 1. Introduction

It is well documented that the fertility of female domestic animals raised at high altitude is reduced in comparison to those raised at low altitude due to the hypoxic environment [1,2]. Although most studies about exposure to high altitudes and hypoxia have focused on its effects on embryonic or fetal losses, intrauterine growth restriction, and birth weight reduction in animals [3], a few recently published papers have shown that reproductive efficiency can be compromised by deficiencies in preovulatory follicle development in sheep raised at high altitude [4] and in mice, as shown using a hypoxic follicular culture system [5].

Female reproduction is reliant on appropriate folliculogenesis and ovulation. Estrogen plays a key role in this process through the expression of nuclear estrogen receptors (ERα and ERβ) in the ovaries [6]. The crucial role of ERα on folliculogenesis and ovulation has been confirmed in vivo, as there is no corpus luteum (CL) in the ovaries of ERα-deficient mice stimulated with human chorionic gonadotropin (hCG) [7]. ERα is also regulated by hypoxia in endometriosis [8], adipose tissues [9], and human embryonic kidney cells [5,10] in different ways, but there are limited studies on the role of hypoxia on ERα in ovarian follicles.

Folliculogenesis is a coordinated process driven by signals from ovarian cells and from the anterior pituitary. It is characterized by an increase in oocyte size and by proliferation and differentiation of the surrounding granulosa cells (GCs) from follicles to antral follicles [5]. However, there are great differences in growth regulation between follicles and antral follicles due to paracrine and endocrine factors, the follicular extracellular matrix, and communication between the follicular compartments (oocyte, granulosa, and theca cells) [6]. Investigating this complex sequence of events has been difficult in vivo because of the limited ability to track or monitor individual follicles. As a result, in vitro ovarian single-follicle culture systems have been developed to provide an important and useful tool for the elucidation of factors involved in the regulation of ovarian function and have potential applications in the areas of infertility, ovarian cancer, and ovarian tissue auto transplantation [5,11,12].

Although the effects of hypoxia on preovulatory follicles have been studied in sheep [1] and mice [5], the exact function and mechanism of hypoxia on follicles development is unclear. The hypothesis of the present study was that hypoxia affects the follicles development at the molecular level. Therefore, during this work, we studied the impact of hypoxia on bovine follicles with a model of in vitro follicles culture.

## 2. Materials and Methods

### 2.1. Isolation and in Vitro Growth of Bovine Follicles

The experiment was approved by the Institutional Animal Care and Use Committee of the Northwest A&F University under permit number 2016ZX08008002. Bovine ovaries (*n* = 106) were collected from adult cows in an abattoir and transported to the laboratory at 30 °C in phosphate buffered saline(PBS)containing penicillin (100 IU/mL), streptomycin (100 mg/mL), and Fungizone (1 mg/mL). Sections of the ovarian cortex were obtained using a scalpel to produce 2 by 2 by 5 mm sections that were washed twice using saline before being transferred onto glass slides in Leibovitz medium (Life Technologies company, Grand Island, NY, USA) supplemented with bovine serum albumin (BSA) (0.5%,W/V,Sigma-Aldrich), penicillin (100 U/mL), and streptomycin (100 μg/mL). Follicles with at least two layers of thecal cells were carefully micro-dissected from the ovarian cortex using 25-gauge needles. The isolated follicles with diameter between 140–150 µm were pooled in four-well plates containing Leibovitz medium, then the culture plates were held in a humidified incubator at 38.5 °C under 5% CO_2_ before they were transferred into 24-well plates. At least 15 follicles of each group were cultured in the plate.

To grow the follicles individually, each follicle was transferred into a single well containing Millipore CM culture plate inserts (Millipore Corporation, Billerica, MA, USA) and 500 µL α-MEM supplemented with follicle stimulating hormone (FSH, 5 IU/mL), insulin (5 µg/mL), transferrin (10 µg/mL), selenium (2 ng/mL), ascorbic acid (20 µg/mL), 0.5% BSA (W/V), penicillin (100 U/mL), and streptomycin (100 µg/mL) in a 24-well plate. Follicles were grown at 38.5 °C under a humidified atmosphere containing 95% air and 5% CO_2_ for six days. On days three and five, seventy percent of the medium was replaced with fresh α-MEM containing the same supplements as described above, with day one being the collection day. Follicles were prevented from attaching to the bottom of the well and led to maintain their intact structure and morphology through the use of Millipore CM culture plate inserts. 

Except noted above, all chemicals and reagents used for follicular culture were purchased from Sigma-Aldrich Corp., Shanghai, China. All experimental procedures were performed in keeping with the guidelines of the Northwest Agricultural & Forestry University Bio-Security Committee. 

### 2.2. Treatment of Follicles Cultured in Vitro

To assess the effects of hypoxia on follicle development, the follicles were divided into four groups on day three. One group of follicles was cultured at 38.5 °C under a humidified atmosphere consisting of 95% air and 5% CO_2_ as the control group (normoxia) [8], the other three groups were experimental groups cultured under hypoxic condition, where follicles were incubated at 38.5 °C under 5% CO_2_ and 1% O_2_ was replaced with N_2_ using a hypoxic chamber (Forma). Starting on day three, follicles were treated using either 10 nm/mL E_2_ (17β-estrodial) or a combination of E_2_ and 1 µM faslodex (ICI 182,780 or ICI, to reverse the activity of an estrogen antagonists), while maintained under hypoxia. To ensure consistent conditions, dimethyl sulfoxide (DMSO) was added into the medium of the normoxia group and the final concentration of DMSO in each well never exceeded 0.1% (V/V). During the process of follicular growth in vitro, the diameters of round follicles (at least 10 follicles) were measured with a dissecting microscope each day until day six to calculate the average follicular growth. 

### 2.3. RNA Extraction, RT-PCR and Real-time PCR

Total RNA was extracted from 20–30 follicles using PicoPure RNA isolation kit (Arcturus Bioscience, Mountain View, CA, USA) according to the manufacturer’s instructions. The cDNA was prepared in a 25 μL reaction containing 1 μg DNase-treated total RNA in the presence of 2 mmol/L oligonucleotide (dT) primer, 5 U OmniscriptRTase, 0.5 mmol/L dideoxynucleotide triphosphate (dNTP) mix and 20 U RNase inhibitor at 37 °C for 1 h. The reaction was terminated at 93 °C for 5 min.

Real-time PCR was performed on an ABI PRISM 7500 sequence detection system (Applied Biosystems, Warrington, UK) with Power SYBR Green PCR Master Mix. The thermal cycling parameters were as follows: 50 °C for 2 min and 95 °C for 10 min (one cycle) and 95 °C for 15 s and 60 °C for 1 min (40 cycles). The specific primers for target genes are listed in Table 1. The samples were run in duplicate and were compared relative to the housekeeping gene β-actin. Identification of the PCR product was verified by melting curve analyses. Quantitative differences in the cDNA target between samples were normalized to a calibrator sample with correction for amplification efficiency. All chemicals and reagents used for Real-time PCR were purchased from QIAGEN Corp., New York, NY, USA.

### 2.4. Western Blot

To extract sufficient protein for western blotting, at least 30 round follicles were collected for the entire experiment. Briefly, after treatment, follicles were transferred into a centrifuge tube and washed three times with cold PBS, resuspended in 500 µL of Radio-Immunoprecipitation Assay (RIPA) lysis buffer(with 10 µL protease inhibitor cocktail and 10 µL PMSF), and centrifuged at 13,000 rpm at 4 °C for 15 min to collect the supernatant. The supernatant protein concentration was measured using the protein assay kit (Thermo Fischer Scientific, Massachusetts, MA, USA). ERα and HIF1A primary and secondary antibodies used for western blotting were purchased from Santa Cruz (Santa Cruz Biotechnology COMPANY, Santa Cruz, CA, USA). Antibodies ERα and HIF1A were raised against β-actin and diluted in PBST containing 5% non-fat milk and membranes were incubated overnight at 4 °C. The membranes were developed with a chemiluminescence detection kit (ECL, Millipore Corporation, Burlington, MA, USA). The optical density of each band was quantified by densitometry. 

### 2.5. Statistical Analysis

All statistical analyses were performed with GraphPad Prism 6 software (GraphPad software Inc., San Diego, CA, USA). Data were transformed to logarithms if they were not normally distributed. ANOVA was used to test the main effects of treatments. Differences between means were tested with the Tukey-Kramer HSD test. Data are presented as mean ± SEM. All experiments were performed with at least three replicates. *p* < 0.05 was considered to represent a significant difference between each pair of groups.

## 3. Results

### 3.1. Effects of Hypoxia on Follicular Growth in Vitro

As shown in Table 2, the selected follicles were categorized into four groups based on their conditions of in vitro culture (Control, Hypoxia, Hypoxia + E_2_, and Hypoxia + E_2_ + ICI). Generally, the follicular diameters increased significantly over the course of 6 days in all groups (*p* < 0.05). From day three to six days, the reduced increase in follicular diameter was significant in both groups of hypoxia and hypoxia + E_2_ + ICI in compared to control group (*p* < 0.05), but no difference was found between the hypoxia and hypoxia + E_2_ + ICI groups (*p* > 0.05). There was no difference found in follicular diameter between the control group and the hypoxia + E_2_ group from day three to five days. Nevertheless, the follicular diameter was significantly smaller in the hypoxia + E_2_ than in control group on day six (*p* < 0.05).

### 3.2. Effects of Hypoxia on Expression of Follicular Growth-Related Genes

**Figure 1:**
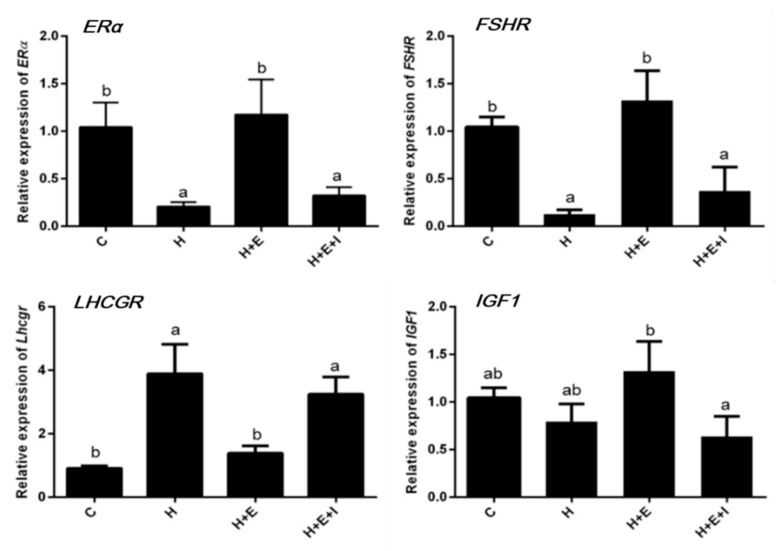
Effects of hypoxia on relative expression of *ERα*, *FSHR*, *LHCGR*, and *IGF1* genes in bovine follicles. C, Control; H, Hypoxia; H + E, Hypoxia with E_2_; and H + E + I, Hypoxia with E_2_ and ICI. Different letters (a,b) on the bar means significant differences (*p* < 0.05).

We quantified the transcript levels of *ERα*, *FSHR*, *LHCGR*, and *IGF1* (Figure 1). As shown in Figure 1, the expression of *ERα* and *FSHR* is significantly downregulated in the hypoxia and hypoxia + E_2_ + ICI groups compared to control group (*p* < 0.05), but no difference in *ERα* and *FSHR* expression was observed between the control and hypoxia + E_2_ groups. The transcription level of *LHCGR* is significantly upregulated in both the hypoxia and hypoxia + E_2_ + ICI groups when compared with the control group (*p* < 0.05). The expression of *IGF1* is significantly downregulated in the hypoxia + E_2_ + ICI group when compared to the hypoxia + E_2_ group (*p* < 0.05).

### 3.3. Effects of Hypoxia on Expression of Hypoxia-Inducible Factor-Related Genes

**Figure 2:**
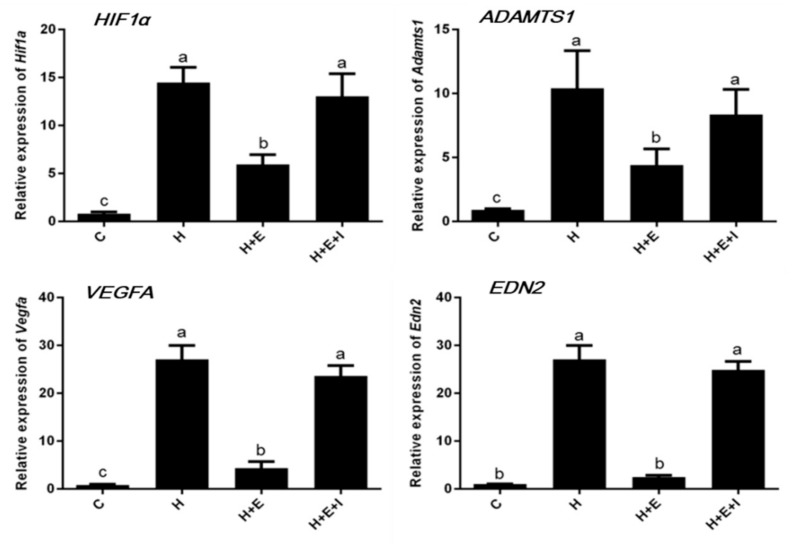
Effects of hypoxia on relative expression of *HIF1A*, *ADAMTS1*, *VEGFA*, and *EDN2* genes in bovine early preantral follicles. C, Control; H, Hypoxia; H + E, Hypoxia with E_2_; and H + E + I, Hypoxia with E_2_ and ICI. Different letters (a–c) on the bar means significant differences (*p* < 0.05).

In a separate experiment, the effects of hypoxia on the transcription levels of *HIF1A* and its downstream genes *ADAMTS1*, *VEGFA*, and *EDN2*, were quantified in cultured bovine ovarian follicles (Figure 2). As shown in Figure 2, these four genes have a similar expression profile. Expression of *HIF1A*, *ADAMTS1*, *VEGFA*, and *EDN2* genes are significantly upregulated in the hypoxia and hypoxia + E_2_ + ICI groups when compared to the control group (*p* < 0.05). Transcription levels of *HIF1A*, *ADAMTS1*, and *VEGFA* genes are significantly higher in the hypoxia + E_2_ group compared to the control group, but lower than those in the hypoxia and hypoxia + E_2_ + ICI groups (*p* < 0.05). 

### 3.4. Effects of Hypoxia on Expression of HIF1A and ERα Proteins

**Figure 3:**
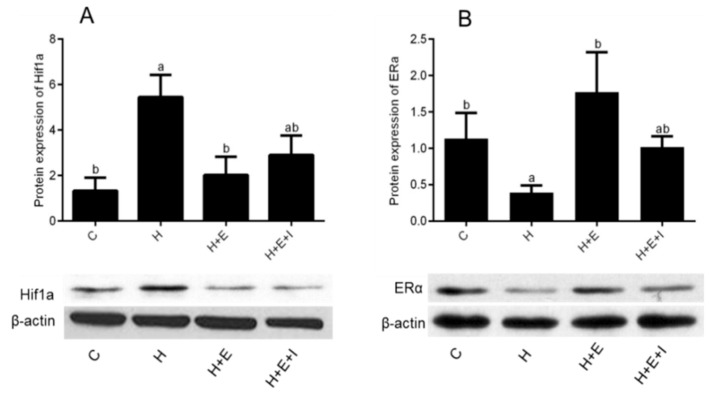
Effects of Hypoxia on the expressions of HIF1A (**A**) and ERα (**B**) proteins in bovine early preantral follicles. C, Control; H, Hypoxia; H + E, Hypoxia with E_2_; H + E + I, Hypoxia with E_2_ and ICI. Different letters (a,b) on the bar means significant differences (*p* < 0.05).

In order to investigate the effect of hypoxia on ERα in an in vitro culture of bovine follicles, the abundance of HIF1A and ERα proteins was measured by western blotting. The results show that hypoxia significantly increases the level of HIF1A protein (Figure 3A) and decreases the level of ERα protein (Figure 3B) in cultured bovine early antral follicles (*p* < 0.05). Under hypoxia, the addition of E_2_ results in a significant decrease of HIF1A and a significantly increase of ERα in cultured follicles (*p* < 0.05). However, the abundance of HIF1A and ERα proteins were not different from the group of E_2_ and ICI added in hypoxic condition compared to the control group.

## 4. Discussions

In the present study, we evaluated the effects of hypoxia on the growth of bovine ovarian follicles using an in vitro follicular growth system and determined the role of hypoxia on ERα in cultured follicles. The parameters that we chose to explore include the percentage of morphologically normal follicles, follicular diameters, the transcription levels of genes related to follicular growth and atresia, and the quantitative expression of ERα and HIF1A proteins. Our results show that growth inhibition of bovine ovarian follicles is mainly induced by a decrease of ERα activity under hypoxic conditions.

In order to obtain normal follicle growth and development in vitro, it is essential to supply adequate oxygen, nutrients, hormones, and growth factors [13,14,15]. Several studies have found that antral follicle growth in vitro is limited by hypoxia [5]. As a major regulatory factor in mammals in maintaining oxygen balance, HIF1A is produced mostly by mammals and humans under anoxic conditions [16], and is detected only during the early stage of corpus luteal formation in the cow, with no measurable expression in later stages of the cycle [17,18]. However, the role of HIF1A in bovine follicles is keeping unclear.

In the present study, our results indicate changes in the growth and quality of bovine early follicles induced by exposure to hypoxia. The bovine follicular diameter is consistently larger under normoxia than follicles cultured under hypoxia (Table 2), suggesting that hypoxia may be a trigger for follicular atresia [19]. The present study also found that bovine follicular growth could be recovered by adding estrogen into the culture medium of the hypoxic group, confirming that follicle growth correlates well with estradiol secretion [20]. In addition, because of the effects of the ERα antagonist, ICI, on the expression of ERα [21], we found that follicular diameters increased more slowly in the hypoxia group co-administered with estrogen and ICI than that of the estrogen-alone hypoxic group. The results presented here suggest that follicle development in vitro under hypoxia is reduced by insufficient ERα function.

Many studies found that hypoxic conditions result in the modulation of growth-related genes in many tissues. In the present study, the transcription of genes associated with follicular development and atresia was investigated to elucidate their expression patterns under hypoxic and normoxic conditions. Transcription of *ERα*, *FSHR*, *LHCGR*, and *IGF1* genes were quantified in bovine ovarian follicles (Figure 1) and the results show that hypoxia significantly downregulates *ERα* and *FSHR* expression in bovine follicles in vitro, but that the downregulation is countered by the addition of estrogen into the follicular culture medium. A possible reason for this different finding may be the difference in species and cell types. *FSHR* is expressed in both immature and mature granulosa cells [22,23] and its expression levels remain constant throughout follicle development at the mRNA and the protein levels [24,25]. However, a downregulation of *FSHR* expression by hypoxia in our study indicates that hypoxia inhibits follicular development and the proliferation of granulosa cells. With the addition of estrogen under hypoxic condition, *FSHR* expression is upregulated in bovine follicles (Figure 1). Furthermore, the expression level of *LHCGR* under hypoxic condition is significantly upregulated in the follicles compared to the controls. In vivo, *LHCGR* is expressed in mature follicles as they approach ovulation, and in response to the luteinizing hormone (LH) surge, *LHCGR* upregulation allows for ovulation and for the luteinization of granulosa cells. Therefore, the expression of *LHCGR* under hypoxic conditions is a sign of granulosa cell luteinization, which is related to follicle shrinking and results in follicular atresia [16]. IGF1 stimulates proliferation and differentiation of the granulosa cells of follicles and *IGF1* expression is regulated by E_2_ through ERα [26]. However, in this work, *IGF1* expression was unchanged in bovine ovarian follicles cultured under hypoxic condition. No difference was observed in the expression of all four above-described genes between the hypoxia group and the hypoxia + E_2_ + ICI group because of the antagonistic effect of ICI on ERα.

In many organs and cells, *HIF1A* gene expression is upregulated under hypoxic conditions and is differentially expressed in the ovary within ovarian cells [27]. We chose to measure the expression of *HIF1A* gene in bovine ovarian follicles in the present study as an indicator of the effects of hypoxia. In agreement with previous studies, we found that increased expression of *HIF1A* is observed in bovine follicles under hypoxic condition, which suggests that a follicle is unable to form an antrum [28]. The link between E_2_/ERα and HIF1A expression has been described in many studies and the results show that ERα is required for estrogen-dependent HIF1α destabilization [20,29]. Our results show that estrogen inhibits *HIF1A* expression in bovine ovarian follicles under hypoxic condition and that this effect is eliminated by ICI. Therefore, we show that ERα plays a crucial role in the downregulatory effects of estrogen on *HIF1A* in cultured follicles under hypoxic condition. As one of the downstream genes of *HIF1A*, the expression of the *ADAMTS1* gene is hormonally regulated in the ovaries by LH and the progesterone receptor, which are involved in many protease cascades that are critical for many biological events, including LH - induced ovulation [30]. The expression of the *ADAMTS1* gene has been well documented in mouse ovulating follicles, and increased expression of *ADAMTS1* is found in endothelial cells under hypoxia [31]. Consistent with previous work, our results show that the expression of by what *ADAMTS1* mRNA is rapidly upregulated in bovine ovarian follicles but downregulated by estrogen addition in hypoxic condition.

It is important to note that hypoxia has been known to induce vascular endothelial growth factor A (VEGFA), an important growth and permeability factor for endothelial cells [32]. *VEGFA* is also highly expressed in the luteinizing GCs and developing CL [33]. Unlike in many other tissues, LH/hCG has been shown to be a major stimulant of *VEGFA* expression in the ovary [32,34]. Consistent with previous findings, hypoxia-induced upregulation of *VEGFA* occurs at the transcriptional level, while downregulation of *VEGFA* takes place in bovine ovarian follicles with the addition of E_2_ under hypoxic condition. 

It has been previously confirmed that endothelin 2 (EDN2) is made by luteinizing GCs as a novel autocrine factor and ablates peptide-modulated GC functions in the bovine ovary [35]. *EDN2* plays an important role in follicular rupture as a regulator of ovulation in the mouse [36]. A recent publication showed that *EDN2* expression in GCs is strongly trigged by hypoxia [9,37]. Hence, we examined the expression of *EDN2* in bovine ovarian follicles and the results reveal that *EDN2* expression is significantly upregulated in the follicles under hypoxia when compared to those under normoxia. 

To investigate the mechanism of hypoxia on developing bovine follicles, we also measured the expression of HIF1A and ERα proteins by western blotting (Figure 3). The expression of Hif1α protein in cultured follicles is generally in accordance with RT-qPCR results, and the outcome confirmed our speculation that the expression of HIF1A protein is increased in bovine ovarian follicles following exposure to hypoxia, which is in agreement with previously published works [10]. Compared with follicles cultured under normoxia, the expression of ERα in follicles under hypoxia is lower, but ERα expression was notably induced by E_2_ even under hypoxia. Weak expression of ERα was detected in the hypoxia + E_2_ + ICI group. These results are in agreement with previous work that shows 17β-estradiol (E_2_) exerts protective effects under hypoxic condition via the downregulation of HIF-1α [38].

## 5. Conclusions

In conclusion, the present study indicates that the growth of bovine follicles is limited under hypoxic conditions due to the inhibition of ERα by hypoxia. However, although the results of this study provide useful information for future studies in this area, further research is required to obtain more information on the molecular mechanism of hypoxia on oocyte growth, proliferation and differentiation of granulosa cells, and signaling pathways of hypoxia on folliculogenesis, as well as to improve the reproductive potential of female animals.

## Figures and Tables

**Table 1:** Bovine specific primers used for real-time PCR.

Gene	Forward Primers (5′–3′)	Reverse Primers (5′–3′)
*β-ACTIN*	ATATCGCTGCGCTGGTCGTC	GCTGCCTCAACACCTCAACCC
*ERα*	AATTCTGACAATCGACGCCAG	GTGCTTCAACATTCTCCCTCCTC
*FSHR*	GCTCCCAATGCAGACCTCTT	CCTCCAGTTTGCAAAGGCAC
*LHCGR*	TGCCTTTGACAACCTCCTCA	AGCATCTGGTTCAGGAGCAC
*IGF1*	CTTGAAGCAGGTGAAGATGCC	AGAGCATCCACCAACTCAGC
*HIF1A*	AGATCTCGGCGAAGCAAAGAGT	CGGCATCCAGAAGTTTTCTCACAC
*ADAMTS1*	TGCTCCAAGACATGCGGCTCAG	TGGTACTGGCTGGCTTCACTTCC
*VEGFA*	CTGTGCAGGCTGCTGTAACG	GTTCCCGAAACCCTGAGGAG
*EDN2*	CTGGCCTGAAGCTGTGGT	ATAGGGGCCAGCCATGAT

**Table 2:** The effects of hypoxia on follicular diameter in vitro.

Items	Day 1	Day 2	Day 3	Day 4	Day 5	Day 6
Control (μm)	145.33 ± 6.53	175.45 ± 7.84 ^A^	238.36 ± 10.67 ^Ba^	281.70 ± 15.55 ^Ca^	316.67 ± 8.59 ^Da^	342.26 ± 11.86 ^Ea^
Hypoxia (μm)	146.26 ± 8.31	173.63 ± 5.48 ^A^	205.48 ± 9.22 ^Bb^	235.87 ± 12.30 ^Cb^	258.16 ± 8.65 ^Db^	276.37 ± 7.52 ^Ec^
Hypoxia + E_2_ (μm)	146.33 ± 4.21	172.34 ± 4.78 ^A^	230.25 ± 7.61 ^Ba^	275.07 ± 10.24 ^Ca^	307.61 ± 9.85 ^Da^	321.66 ± 8.11 ^Eb^
Hypoxia + E_2_ + ICI (μm)	146.26 ± 8.31	174.36 ± 8.45 ^A^	211.36 ± 2.92 ^Bb^	240.78 ± 3.12 ^Cb^	261.65 ± 6.81 ^Db^	281.67 ± 5.27 ^Ec^

Data are means ± SEM. of three independent replicates. Values within a row without a common superscript (A–E) indicated significant differences (*p* < 0.05). Values within a column without a common superscript (a–b) indicated differences (*p* < 0.05).

## References

[1] Parraguez V.C., Atlagich M., Diaz R., Bruzzone M.E., Behn C., Raggi L.A. (2005). Effect of hypobaric hypoxia on lamb intrauterine growth: Comparison between high- and low-altitude native ewes. Reprod. Fertil. Dev..

[2] Parraguez V.H., Atlagich M., Díaz R., Cepeda R., González C., De L.R.M., Bruzzone M.E., Behn C., Raggi L.A. (2006). Ovine placenta at high altitudes: Comparison of animals with different times of adaptation to hypoxic environment. Anim. Reprod. Sci..

[3] Hartinger S., Tapia V., Carrillo C., Bejarano L., Gonzales G.F. (2006). Birth weight at high altitudes in Peru. Int. J. Gynecol. Obstet..

[4] Parraguez V.H., Diaz F., Cofre E., Urquieta B., De Los R.M., Astiz S., Gonzalez-Bulnes A. (2014). Fertility of a high-altitude sheep model is compromised by deficiencies in both preovulatory follicle development and plasma LH availability. Reprod. Domest. Anim..

[5] Connolly J.M., Kane M.T., Quinlan L.R., Dockery P., Hynes A.C. (2017). Corrigendum to: Hypoxia limits mouse follicle growth in vitro. Reprod. Fertil. Dev..

[6] Korach K.S. (1994). Insights from the study of animals lacking functional estrogen receptor. Science.

[7] Krege J.H., Hodgin J.B., Couse J.F., Enmark E., Warner M., Mahler J.F., Sar M., Korach K.S., Gustafsson J.A., Smithies O. (1998). Generation and reproductive phenotypes of mice lacking estrogen receptor beta. Proc. Natl. Acad. Sci. USA.

[8] Wu M.H., Lu C.W., Chang F.M., Tsai S.J. (2012). Estrogen receptor expression affected by hypoxia inducible factor-1alpha in stromal cells from patients with endometriosis. Taiwan J. Obstet. Gynecol..

[9] Kim J., Bagchi I.C., Bagchi M.K. (2009). Signaling by hypoxia-inducible factors is critical for ovulation in mice. Endocrinology.

[10] Kim M., Neinast M.D., Frank A.P., Sun K., Park J., Zehr J.A., Vishvanath L., Morselli E., Amelotte M., Palmer B.F. (2014). ERα upregulates Phd3 to ameliorate HIF-1 induced fibrosis and inflammation in adipose tissue. Mol. Metab..

[11] Xu M., Barrett S.L., West-Farrell E., Kondapalli L.A., Kiesewetter S.E., Shea L.D., Woodruff T.K. (2009). In vitro grown human ovarian follicles from cancer patients support oocyte growth. Hum. Reprod..

[12] West E.R., Zelinski M.B., Kondapalli L.A., Gracia C., Chang J., Coutifaris C., Critser J., Stouffer R.L., Shea L.D., Woodruff T.K. (2009). Preserving female fertility following cancer treatment: Current options and future possibilities. Pediatr. Blood Cancer.

[13] Picton H.M., Harris S.E., Muruvi W., Chambers E.L. (2008). The in vitro growth and maturation of follicles. Reproduction.

[14] Smitz J.E., Cortvrindt R.G. (2002). The earliest stages of folliculogenesis in vitro. Reproduction.

[15] Telfer E.E., Zelinski M.B. (2013). Ovarian follicle culture: Advances and challenges for human and nonhuman primates. Fertil. Steril..

[16] Thompson J.G., Brown H.M., Kind K.L., Russell D.L. (2015). The Ovarian Antral Follicle: Living on the Edge of Hypoxia or Not?. Biol. Reprod..

[17] Nishimura R., Okuda K. (2010). Hypoxia is important for establishing vascularization during corpus luteum formation in cattle. J. Reprod. Dev..

[18] Wang G.L., Jiang B.H., Rue E.A., Semenza G.L. (1995). Hypoxia-inducible factor 1 is a basic-helix-loop-helix-PAS heterodimer regulated by cellular O_2_ tension. Proc. Natl. Acad. Sci. USA.

[19] Spears N., Boland N.I., Murray A.A., Gosden R.G. (1994). Mouse oocytes derived from in vitro grown primary ovarian follicles are fertile. Hum. Reprod..

[20] Wycherley G., Downey D., Kane M.T., Hynes A.C. (2004). A novel follicle culture system markedly increases follicle volume, cell number and oestradiol secretion. Reproduction.

[21] Arao Y., Hamilton K.J., Ray M.K., Scott G., Mishina Y., Korach K.S. (2011). Estrogen receptor alpha AF-2 mutation results in antagonist reversal and reveals tissue selective function of estrogen receptor modulators. Proc. Natl. Acad. Sci. USA.

[22] Jungyoon C., Dukkyung K., Seungki L., Youngjoo L. (2005). Cobalt chloride-induced estrogen receptor alpha down-regulation involves hypoxia-inducible factor-1alpha in MCF-7 human breast cancer cells. Mol. Endocrinol..

[23] Yi J.M., Kwon H.Y., Cho J.Y., Lee Y.J. (2009). Estrogen and hypoxia regulate estrogen receptor alpha in a synergistic manner. Biochem. Biophys. Res. Commun..

[24] Kind K.L., Banwell K.M., Gebhardt K.M., Macpherson A., Gauld A., Russell D.L., Thompson J.G. (2013). Microarray analysis of mRNA from cumulus cells following in vivo or in vitro maturation of mouse cumulus-oocyte complexes. Reprod. Fertil. Dev..

[25] Brown H.M., Anastasi M.R., Frank L.A., Kind K.L., Richani D., Robker R.L., Russell D.L., Gilchrist R.B., Thompson J.G. (2015). Hemoglobin: A gas transport molecule that is hormonally regulated in the ovarian follicle in mice and humans. Biol. Reprod..

[26] Watson C.S., Jeng Y.J., Guptarak J. (2011). Endocrine disruption via estrogen receptors that participate in nongenomic signaling pathways. J. Steroid Biochem. Mol. Biol..

[27] Boonyaprakob U., Gadsby J.E., Hedgpeth V., Routh P.A., Almond G.W. (2005). Expression and localization of hypoxia inducible factor-1alpha mRNA in the porcine ovary. Can. J. Vet. Res..

[28] West-Farrell E.R., Xu M., Gomberg M.A., Chow Y.H., Woodruff T.K., Shea L.D. (2009). The mouse follicle microenvironment regulates antrum formation and steroid production: Alterations in gene expression profiles. Biol. Reprod..

[29] Xu J., Lawson M.S., Yeoman R.R., Pau K.Y., Barrett S.L., Zelinski M.B., Stouffer R.L. (2011). Secondary follicle growth and oocyte maturation during encapsulated three-dimensional culture in rhesus monkeys: Effects of gonadotrophins, oxygen and fetuin. Reproduction.

[30] Richards J.S., Hernandez-Gonzalez I., Gonzalez-Robayna I., Teuling E., Lo Y., Boerboom D., Falender A.E., Doyle K.H., LeBaron R.G., Thompson V. (2005). Regulated expression of ADAMTS family members in follicles and cumulus oocyte complexes: Evidence for specific and redundant patterns during ovulation. Biol. Reprod..

[31] Hatipoglu O.F., Hirohata S., Cilek M.Z., Ogawa H., Miyoshi T., Obika M., Demircan K., Shinohata R., Kusachi S., Ninomiya Y. (2009). *ADAMTS1* is a unique hypoxic early response gene expressed by endothelial cells. J. Biol. Chem..

[32] Rico C., Dodelet Devillers A., Paquet M., Tsoi M., Lapointe E., Carmeliet P., Boerboom D. (2014). HIF1 Activity in Granulosa Cells Is Required for FSH-Regulated *VEGFA* Expression and Follicle Survival in Mice1. Biol. Reprod..

[33] Zhang Y., Wang S.F., Zheng J.D., Zhao C.B., Zhang Y.N., Liu L.L., Huang J.H. (2016). Effects of testosterone on the expression levels of AMH, VEGF and HIF-1α in mouse granulosa cells. Exp. Ther. Med..

[34] De Francesco E.M., Lappano R., Santolla M.F., Marsico S., Caruso A., Maggiolini M. (2013). HIF-1α/GPER signaling mediates the expression of VEGF induced by hypoxia in breast cancer associated fibroblasts (CAFs). Breast Cancer Res..

[35] Klipper E., Levit A., Mastich Y., Berisha B., Schams D., Meidan R. (2010). Induction of endothelin-2 expression by luteinizing hormone and hypoxia: Possible role in bovine corpus luteum formation. Endocrinology.

[36] Yalu R., Oyesiji A.E., Eisenberg I., Imbar T., Meidan R. (2015). HIF1A-dependent increase in endothelin 2 levels in granulosa cells: Role of hypoxia, LH/cAMP, and reactive oxygen species. Reproduction.

[37] Na G., Bridges P.J., Koo Y., Ko C. (2008). Role of hypoxia in the regulation of periovulatory *EDN2* expression in the mouse. Can. J. Physiol. Pharm..

[38] Li Y., Liu Y., Lu Y., Zhao B. (2017). Inhibitory effects of 17β-estradiol or a resveratrol dimer on hypoxia-inducible factor-1α in genioglossus myoblasts: Involvement of ERα and its downstream p38 MAPK pathways. Int. J. Mol. Med..

